# A multifaceted strategy to improve recombinant expression and structural characterisation of a *Trypanosoma* invariant surface protein

**DOI:** 10.1038/s41598-022-16958-x

**Published:** 2022-07-26

**Authors:** Hagen Sülzen, Jitka Votrubova, Arun Dhillon, Sebastian Zoll

**Affiliations:** 1 Institute of Organic Chemistry and Biochemistry, Academy of Sciences of the Czech Republic, Flemingovo namesti 2, 16610 Prague 6, Czech Republic; 2 Faculty of Science, Charles University, Albertov 6, 12800 Prague 2, Czech Republic

**Keywords:** Expression systems, Protein purification, Protein design

## Abstract

Identification of a protein minimal fragment amenable to crystallisation can be time- and labour intensive especially if large amounts are required and the protein has a complex fold and functionally important post-translational modifications. In addition, a lack of homologues and structural information can further complicate the design of a minimal expression construct. Recombinant expression in *E. coli* promises high yields, low costs and fast turnover times, but falls short for many extracellular, eukaryotic proteins. Eukaryotic expression systems provide an alternative but are costly, slow and require special handling and equipment. Using a member of a structurally uncharacterized, eukaryotic receptor family as an example we employ hydrogen–deuterium exchange mass spectrometry (HDX-MS) guided construct design in conjunction with truncation scanning and targeted expression host switching to identify a minimal expression construct that can be produced with high yields and moderate costs.

## Introduction

Recombinant protein expression in a heterologous system highly depends on precise prediction of secondary structure boundaries to yield soluble protein. Soluble expression however is no guarantor for successful crystallisation of a protein as the presence of flexible, low-complexity regions at the termini can prevent the formation of a highly ordered crystal lattice necessary for high-resolution diffraction. The need to identify a minimal domain amenable to crystallisation therefore calls for mapping the secondary structure landscape of a target protein with the highest possible precision.

Secondary structure prediction has been extensively studied. It has seen many improvements since its inception^1–3^ which renders it still the most commonly used method for the design of recombinant expression constructs. Prediction accuracy hereby depends on the chemo-physical properties of amino acids in a sequence as well as the evolutionary relationships of a protein, such as homology to proteins of known structure. Together with ever growing archives (e.g. Protein Data Bank, PDB) of experimentally determined structures, current algorithms reach an accuracy of 80–90%^4,5^. Lack of significant homology to other structurally characterised protein families however has an adverse effect, resulting in low confidence of the prediction. Recently AlphaFold, an AI system developed by DeepMind and EMBL-EBI has entered a developmental stage which does not only allow prediction of secondary but also tertiary structures of proteins with hitherto unprecedented accuracy, using a novel deep learning network^6^. This has also been reported for proteins adopting novel folds, i.e. in absence of structural homologies that could otherwise aid prediction. Examples of accurate novel fold predictions, such as proteins from extremophiles, are however still scarce and therefore require additional validation. Furthermore, prediction accuracy for secondary structure boundaries is lower than for their internal regions as the number of possible secondary structure elements is more conserved than their length^5^. Consequently, secondary structure prediction of proteins from isolated families with no structural homologues and functional assignment has to be aided by experimental data to achieve the highest possible accuracy.

The production of soluble protein alone, however, is not the only concern in recombinant protein expression. The protein of interest also has to be functional and to be produced in quantities sufficient for downstream applications such as functional studies (e.g. Isothermal titration calorimetry), animal immunisations for antibody generation (e.g. Llamas), or structural studies (e.g. NMR and crystallisation)—some of which require considerable amounts of highly pure protein.

Traditionally, *Escherichia coli* is used for high yield expression, but due to its basic procaryotic folding machinery, its usefulness is often limited to intracellular proteins with less complex folds. Lack of glycosylation and improperly formed or absent disulphide bonds can impair or abrogate the function of extracellular proteins of eukaryotic origin when expressed in *E. coli*. Mammalian and insect cells are the most common expression systems of choice for such proteins. An alternative is the *Leishmania* expression system (Lexsy). In recent years many such systems have been commercialised as kits, allowing for scalable and reproducible production of even challenging proteins. Testing dozens of constructs however, particularly in mammalian cells, is, even in the case of transient expressions, expensive (media, transfection reagents, plastics) and time consuming (growth time, contaminations). Additionally, laboratories often lack the necessary infrastructure and expertise. Due to shorter doubling times Lexsy represents an alternative to insect cells, but it does not offer the same spectrum and variety of strains, transfection systems and expression media (e.g. serum-free media) as insect cell systems.

To overcome the described challenges, we propose a hybrid approach for the identification of a minimal expression construct that lacks extended low-complexity regions at the termini. We employ a combination of hydrogen deuterium exchange mass spectrometry (HDX-MS) and truncation scanning together with initial expression screening in *E. coli* and final high-yield production of the identified domain in insect cells.

We use ISG65 (Invariant surface glycoproteins 65) from the eukaryotic parasite *Trypanosoma brucei* as an example. Invariant surface glycoproteins, type I transmembrane proteins, have been discovered about 30 years ago^7^ and since then have been the subject of numerous studies^8–13^. Nevertheless, despite many efforts, no structure of an ISG has been determined yet. This might in part be related to difficulties in construct design. While the expression constructs of crystallised GPI(glycosylphosphatidylinositol)-anchored trypanosome surface proteins terminated at the position of the omega site (GPI-anchor attachment site)^14–18^, the same straightforward approach for determination of the C-terminal domain boundaries appears not to be applicable to the group of surface proteins harbouring single pass transmembrane domains (TMDs), as shown by ISG65 as an example.

We believe that the strategy outlined here could serve as a template for expression and structure determination of challenging proteins by using established technologies and combining them in a novel, targeted manner that effectively avoids time-consuming trial and error approaches for construct screening.

## Results

### HDX-MS as a tool to identify low-complexity regions in proteins

**Figure 1:**
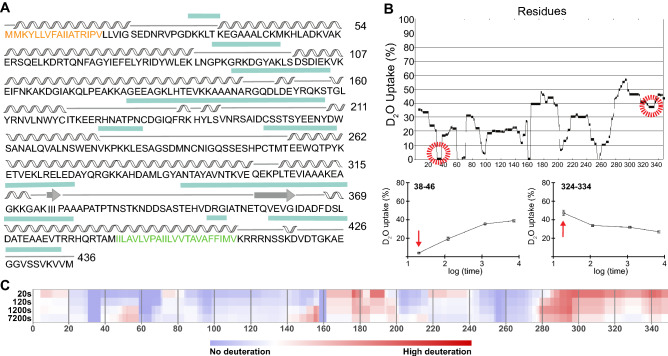
Mapping of ISG65 low-complexity regions. (**A**) Secondary structure prediction of ISG65 adapted from PSIPred^19,20^. Helices are depicted as wavy lines, beta strands as arrows and disordered sections as straight lines. Regions with a low confidence score are marked with a turquoise box above the sequence. Positions of signal-peptide and transmembrane domain are depicted in orange and green, respectively. (**B**) Uptake plot showing the incorporation of deuterium across the sequence of ISG65 20 s after start of the deuteration reaction (main panel). Selected peptides showing the rates of deuterium uptake over time representative for an ordered (bottom left) or disordered (bottom right) region. Red arrows mark the 20 s timepoint. The HDX-MS experiment was performed in triplicates. (**C**) Chiclet plot illustrating the relative deuteration across the sequence of ISG65 at 20, 120, 1200 and 7200 s of deuteration, adapted from MSTools^28^. The amount of relative deuteration is gradually colour coded from blue (no deuteration) to red (high deuteration).

No homologues are known for the family of invariant surface glycoproteins. Secondary structure prediction for ISG65 using PsiPred^19,20^ shows a low confidence score across the whole sequence and, particularly, in the C-terminal region (Fig. 1A). In a first approach, an initial expression construct comprising the whole extracellular domain (ECD), truncated at the position of the TMD and lacking the N-terminal signal-peptide (ISG65_18-386_), was chosen for expression in *E. coli* T7 shuffle cells to promote disulphide bond formation in the cytoplasm. This construct failed to express, likely due to inaccuracies in the prediction of the transmembrane domain (TMD). A second construct with the new C-terminus further away from the TMD (ISG65_18-363_) could however be produced successfully. To rationalise and accelerate the identification of a minimal construct optimised for crystallisation, we set out to guide our efforts by experimental data using HDX-MS that can be used to identify regions of low complexity that have faster rates of deuteration compared to well-structured regions^21^. Prior to MS analysis, proteolytic digestion of ISG65 was optimised to achieve maximum coverage of the protein sequence with many overlapping peptides (97%, Supplementary Figure 1). This resulted in high resolution mapping of deuterated regions.

The deuteration plot shows a steady increase in deuteration rate starting from residue 260 of ISG65 (Fig. 1B,C; Supplementary Figure 2). Uptake rates plateau at residue 300 and remain high towards the C-terminus of the protein with the exception of a trough approximately around residue 330. This points to the presence of lower secondary structure content in the C-terminal, the membrane-proximal part of the protein.

At the N-terminus deuteration declines rapidly starting from the end of the signal peptide at residue 20 until it reaches almost zero between residues 30–40, indicative for the beginning of a well-structured region which is in line with the secondary structure prediction and position of the signal peptide. Using HDX-MS analysis to approximate a well-expressing, minimal construct amenable to crystallization, we could readily establish the new N-terminus. The precise C-terminal end of the minimal expression construct however was less obvious due to a slower transition to higher deuteration rates in comparison to the N-terminal region and the presence of a local minimum (residues 320–330).

### Truncation scanning established the C-terminal domain boundaries

An initial expression trial with a construct truncated at residue 270 at the end of a well-structured region as determined by HDX-MS did not result in soluble expression. Pursuing our aim to generate a minimal construct for crystallisation we therefore decided to use residue 330 within the local minimum of a less structured region as a starting point for a series of constructs with the same N-terminus and sequentially shortened C-termini (Fig. 1B). For truncation scanning a stop codon was introduced every 2 residues. All constructs were expressed in *E. coli* and purified by IMAC using the same methods as for the longer variants. The final protein yields were further normalized to the cell mass before lysis.

**Figure 2:**
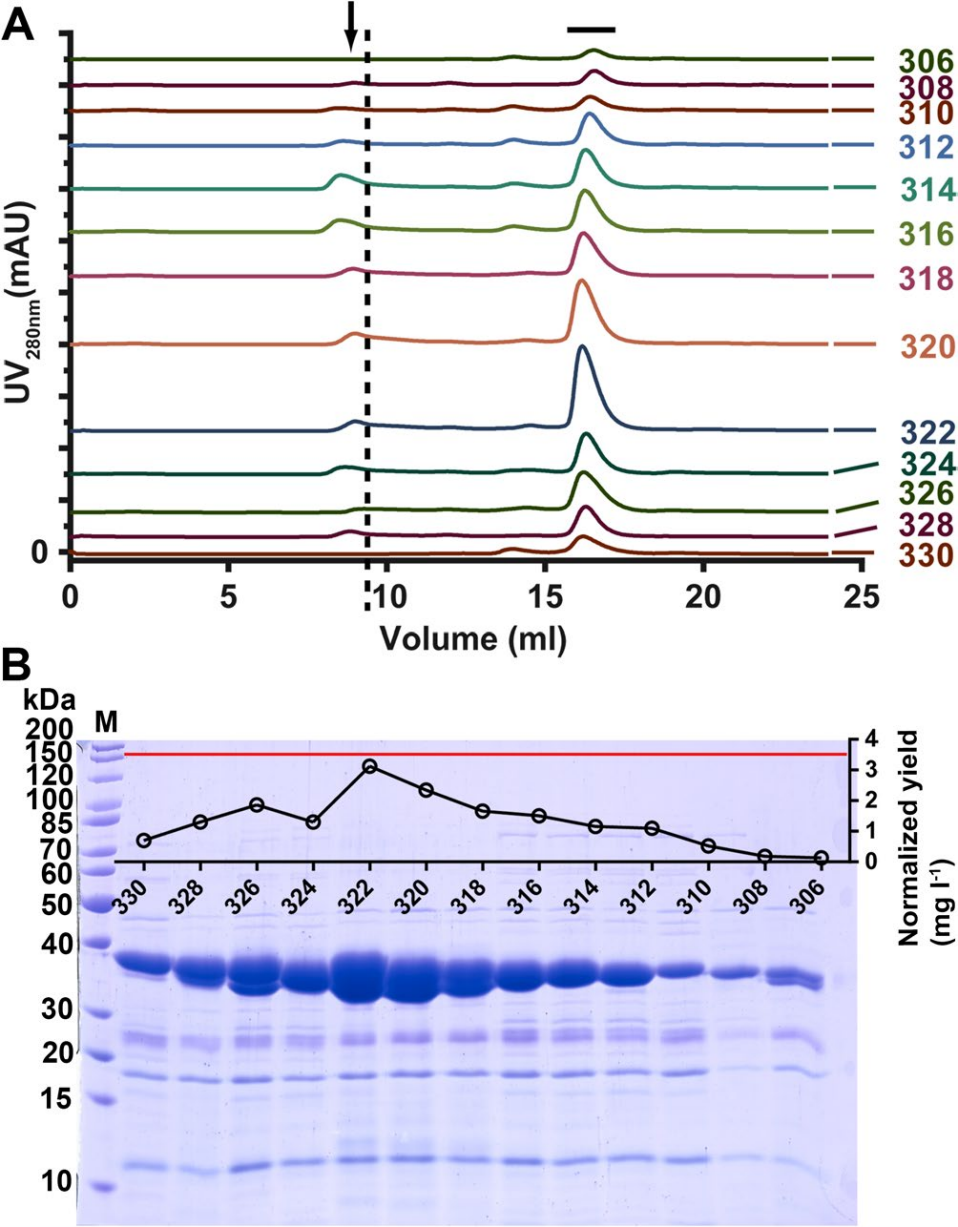
Yields of *E. coli* expressed ISG65 decrease with construct length. (**A**) Stacked size exclusion chromatograms of all ISG65 constructs expressed in *E. coli*. Major tick marks correspond to 300 mAU. Numbers indicate the position of the C-terminal truncation. Peaks corresponding to correctly folded ISG65 are indicated by a black horizontal bar, aggregates by an arrow. The void volume of the column is marked by a dashed, vertical line. (**B**) SDS-PAGE analysis of SEC peak fractions. Total protein yields were calculated for each peak by UV_280nm_ absorption and were normalized to the cell mass in 1 L of culture. All edges of the gel, except the left side, are digitally cropped for clarity. Displayed is the whole separating gel as indicated by the lowest and the highest molecular weight bands of the marker (M).

The total yields of expressed and purified protein generally declined with successive shorting of the C-terminus (Fig. 2). Purifications also contained aggregates of the target protein that eluted in the void volume of the size exclusion column. This further diminished already low yields, but also pointed to general problems of the expression host producing correctly folded protein. The shortest construct resulting in expression of soluble protein was ISG65_32-306_. Expression could no longer be observed in even shorter constructs, suggesting that the C-terminal domain boundary had been identified.

Expression, however, was weak, resulting in exceedingly low yields of soluble protein (< 0.1 mg per litre of culture) after 2 purification steps (Fig. 2). Furthermore, the purified protein still contained significant amounts of contaminations originating from expression in the *E. coli* cytoplasm that would necessitate additional purification steps to establish > 95% purity.

### Expression host switching boosts yield of minimal construct 250-fold

Reasoning that resolute shorting of the construct might have further challenged the *E. coli* folding machinery and thus resulted in lower expression rates we switched to a eukaryotic system for expression of the minimal construct. Furthermore, we decided to include a signal sequence for the secretory pathway in order to facilitate folding in the non-reducing environment of the culture medium. The expression system of choice were insect cells because of their easy handling and lack of requirements for expensive equipment (such as CO_2_ incubators and shakers).

Protein purity was further increased through usage of serum-free media containing less contaminating proteins in combination with utilisation of a C-tag for affinity purification which was previously reported to improve expression yields for a *Plasmodium* surface protein^22^ and is short enough to be unlikely to impair crystallisation.

**Figure 3:**
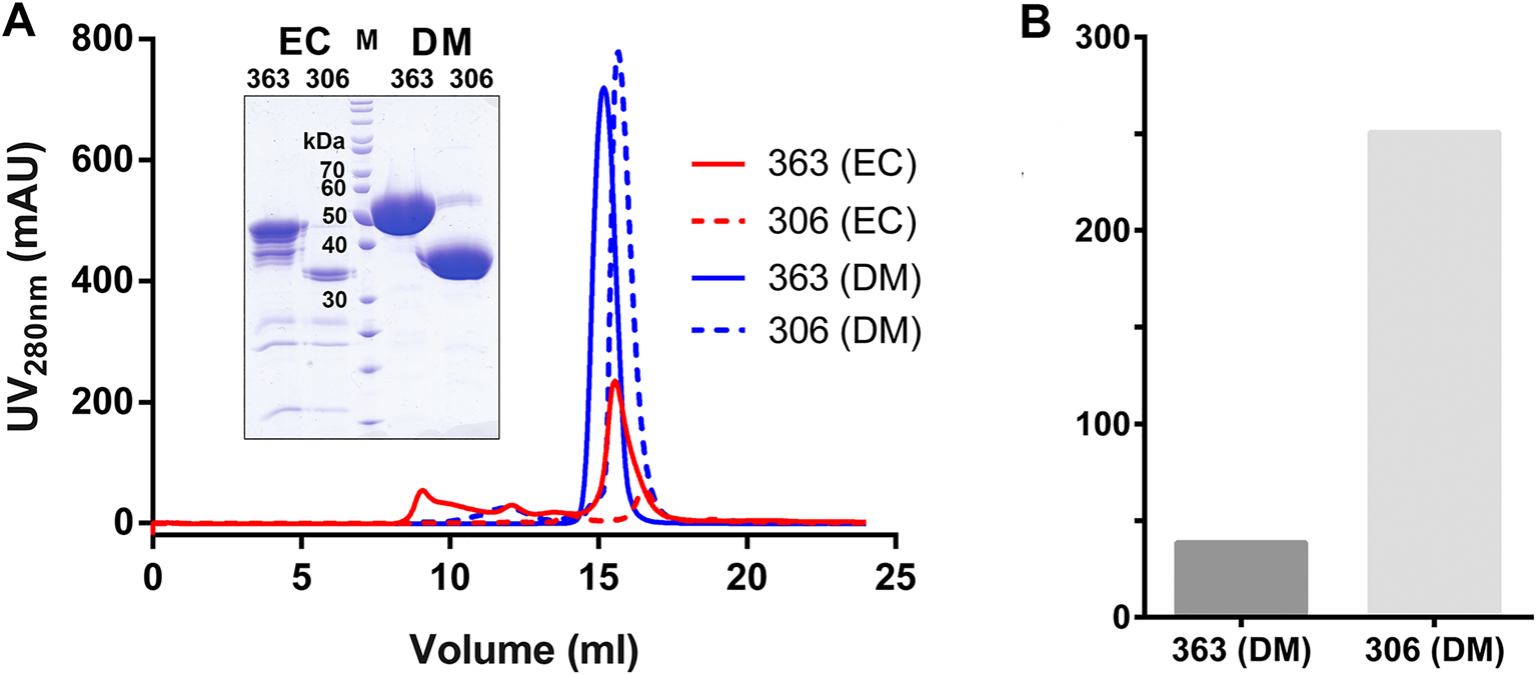
Comparison of recombinant expression yields in *E. coli* and insect cells. Expression in S2 cells results in higher yields, purity and shows no correlation with construct length. (**A**) Size exclusion chromatograms and SDS-gel (inset) of shortest (dashed line) and longest (full line) ISG65 constructs expressed in *E. coli* and insect cells. Bacterial culture volumes were tenfold higher. The protein gel was digitally cropped for clarity. The complete gel is shown in Supplementary Figure 3. (**B**) Expression yields normalised to 1 L of cell culture. Normalised yields were 38-fold (longest construct, dark grey), and, respectively, 250-fold higher (shortest construct, light grey) for the same constructs expressed in insect cells. EC, *E. coli*; DM *Drosophila melanogaster;* M, Marker.

Expression in insect cells, for both the longest (ISG65_32-363_) as well as the shortest (ISG65_32-306_) construct, yielded approx. 30 mg of highly pure protein per litre of culture after 2 purification steps, exceeding the yields achieved in *E. coli* by approx. 40–250-fold (Fig. 3, Supplementary Figure 3). It is noteworthy that unlike in *E. coli*, the minimal construct showed the same expression level as the full-length protein in insect cells. Moreover, no aggregates of either protein could be detected in SEC. The small shift in retention times compared to the same proteins expressed in *E. coli* can be attributed to the presence of posttranslational modifications that alter the hydrodynamic radius (Fig. 3).

### Experimental identification of domain boundaries facilitates structure prediction

**Figure 4:**
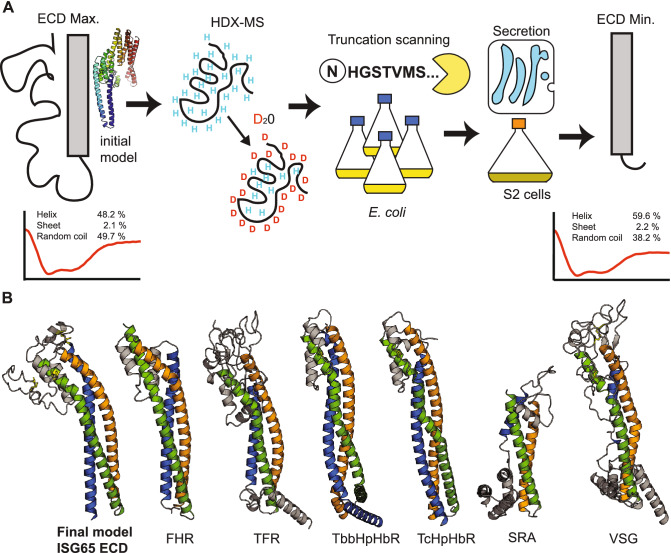
Flow-chart for rational construct design and guided model generation. (**A**) Simplified workflow for identification of a minimal construct that can be produced with high yields and purity. The longest ISG65 construct has a high random coil content (CD measurement, representative curve) which results in generation of an unrealistic model. The identified minimal construct of ISG65 has > 10% lower random coil content according to CD analysis (representative curve below). (**B**) Omission of the identified low-complexity region results in generation of a more realistic ISG65 model (far left). This model shows the same three-helix bundle that is canonical for structurally characterised, GPI-anchored trypanosoma surface proteins ([left to right] PDB IDs: 6XZ6, 6SOY, 4X0J, 4E40, 6ELU, 1VSG). ISG65 ECD, invariant surface glycoprotein 65 extracellular domain; FHR, factor H receptor; TFR, transferrin receptor; TbbHpHbR, *Trypanosoma brucei brucei* haptoglobin-hemoglobin receptor; Tc, *Trypanosoma congolense*; SRA, serum-resistance associated protein; VSG, Variant surface glycoprotein.

In summary we present an integrated strategy for HDX-MS assisted domain identification and high-yield expression of an extracellular eukaryotic protein that can easily be adapted for other challenging proteins. Figure 4A provides an overview of the workflow. While HDX-MS is useful to identify the general region of disorder within a protein, especially at the termini, the method is limited in resolution by the lengths of the obtained peptides (Supplementary Figure 1). HDX-MS does, however, provide a starting point for truncation scanning to determine the domain boundaries more precisely. This step is very time consuming and therefore recommended to be carried out in *E. coli*. Once a minimal construct has been determined, expression can be switched over to insect cells, a moderately expensive and fast eukaryotic system for high yield and high purity production of the target protein (Fig. 4A).

A precise and careful experimental determination of domain boundaries within a protein can compensate for the lack of structural templates in fold prediction and help to generate a more realistic model. Using iTASSER^23^, the final model of ISG65 was calculated omitting the long, low-complexity region at the C-terminus of the sequence. This model shows well-defined secondary structure and is, in contrast to the initial model, in agreement with the general fold of other trypanosome surface proteins despite having < 20% sequence identity to the closest structural homologue (Fig. 4B).

The presence of a low-complexity region in the C-terminal domain of ISG65, as identified by HDX-MS, is corroborated by circular dichroism (CD) spectroscopy performed on both the longest as well as the shortest ISG65 construct. CD analysis revealed that the random coil contribution to the overall secondary structure content is reduced by more than 10% in the minimal construct of ISG65 (Fig. 4A) while the melting temperature remained unchanged (Supplementary Figure 4).

## Discussion

The multifaceted strategy outlined in this study combines the advantages of bacterial and eukaryotic expression systems for recombinant production of complex eukaryotic proteins.

Particularly proteins with glycans and multiple disulphide bonds do not present an easy target for heterologous expression in *E. coli*. The use of T7 shuffle cells or similar BL21 derivatives that promote disulphide formation can alleviate the limitations of cytoplasmic expression, but reduced growth rates as compared to standard expression cells diminish yields. Additionally, we consistently found a certain fraction of the soluble, IMAC-purified protein to form aggregates which eluted in the void volume of the gel filtration column. This further decreased the overall amount of functional protein and might be the consequence of incomplete disulphide formation which has been reported previously^24^.

Generally, it was possible to produce the target protein in *E. coli,* but correct folding in conjunction with high yields seemed to pose a challenge for the basic prokaryotic folding machinery. In *E. coli,* unlike in insect cells, the expression levels differed drastically between the longest and shortest construct. More remarkably, when comparing the overall yields only for the shortest construct we observed a several 100-fold increase in S2 cells over *E. coli*. This suggests that the truncation of an already complex fold has further challenged the bacterial folding machinery to the point where hardly any protein can be produced anymore. Production of the minimal construct in insect cells on the other hand was not affected by truncation, resulting in yields comparable to the longer construct. Furthermore, both *E. coli* produced ISG65_18-363_ and S2 cell produced ISG65_32-306_ show the same melting temperatures. This implies that the structural integrity of the overall fold was not compromised by removal of the terminal regions and that the differences in expression levels are likely to originate in in the differential efficiency of the two systems to produce a complex eukaryotic protein. Our study, using a structurally uncharacterised surface receptor of a eukaryotic parasite as an example, shows that soluble expression in *E. coli* is no guarantor for high protein yields and that weak expression in a bacterial system is not necessarily an indicator for incorrectly chosen domain boundaries.

The initial model calculated for the extracellular domain of ISG65, using the popular prediction program iTASSER, did not resemble the fold of any known trypanosoma surface protein. Especially the C-terminal domain was modelled rather unrealistic without any connection to the rest of the molecule and an unexpectedly high helical content due to a low confidence score in this region. Experimentally identifying low-complexity regions and excluding them from model building could serve to focus the prediction on the structurally conserved core-region of the protein, thus resulting in a more realistic model. In our case this strategy revealed a fold that is highly similar to the canonical 3-helix bundle fold of structurally known surface receptors^14–18,25^. This structural homology was unexpected and could have neither been inferred from the sequence alone nor from the initial model. This increases confidence in the overall correctness of the predicted model which, for example, could serve as a molecular replacement search model in crystal structure determination. Interestingly, a new AlphaFold2-based resource adapted for *Trypanosoma* and *Leishmania* proteins^26^ yielded an initial model that already showed a 3-helix bundle fold, albeit with an unusual arrangement of alpha-helices and long disordered sections (Supplementary Figure 5) in the C-terminal region. However, more reference structures of *Trypanosoma* transmembrane proteins are needed to judge the general validity of our approach as well as the accuracy of predicted models without homologies.

## Material and methods

### Recombinant expression and purification of ISG65

#### *Escherichia coli*

A gene fragment coding for the extracellular domain of ISG65 (*Trypanosoma brucei gambiense* DAL972, gene ID 23,858,558) comprising residues 18-363 was cloned into the bacterial expression vector pET15b (EMD Millipore). Further constructs were truncated N-terminally (residue 32) as well as C-terminally (residues 330–303). All constructs contained a N-terminal hexa-histidine tag. C-terminal truncations were carried out by introducing a stop codon in a 2-residue window using the Q5 site-directed mutagenesis kit (New England Biolabs). Expression constructs were introduced in *E. coli* T7 shuffle cells (New England Biolabs) by heat-shock transformation. Protein expression was induced by addition of 1 mM isopropyl-β-D-thiogalacto-pyranoside (IPTG) and carried out for 16 h at 22 °C. Cells were sedimented at 6,000 g for 15 min and pellets weighed for normalisation of protein expression levels. The cell pellets were resuspended in ice-cold buffer A (20 mM Tris pH8, 150 mM NaCl, 10 mM imidazole) and lysed by sonication. Temperature during sonication was monitored and did not exceed 20 °C. Following cell lysis, supernatant was cleared by centrifugation (50,000 g, 1 h, 4 °C). Soluble protein was purified by immobilized metal affinity chromatography (IMAC) using a 1 ml HisTrap HP on ÄKTA start (GE). Immobilized, His-tagged protein was washed with buffer B (20 mM Tris pH8, 150 mM NaCl, 20 mM imidazole) and eluted from the column with buffer C (20 mM Tris pH8, 150 mM NaCl, 500 mM imidazole). Eluate was dialysed against buffer D (20 mM HEPES pH7.5, 150 mM NaCl) for 16 h at 4 °C. The dialysed protein solution was concentrated by ultrafiltration (Amicon Ultra, EMD Millipore, molecular weight cut-off, 10,000 Da) and further purified by size exclusion chromatography (SEC) in buffer D using a Superdex 200 Increase 10/300 GL on ÄKTA pure (GE). SEC peak fractions were analysed for presence and purity of the target protein using SDS-PAGE.

#### Insect cells

For expression in *Drosophila melanogaster* S2 cells, ISG65 gene fragments encoding residues 18-363 and 32-306 were cloned into the pExpress2.1 vector (ExpreS2ion Biotechnologies). Recombinant ISG65 comprising an N-terminal secretion signal and a C-terminal C-tag was produced following manufacturer’s recommendations (ExpreS2ion Biotechnologies). Briefly, 5 ml of 2 × 10^6^ cells/ml, cultured in Ex-cell 420 serum free medium (14420C, SAFC Biosciences), were transfected with 0.25 ml of 1 × lipofectamine-based reagent (ExpreS2ion Biotechnologies) and 12.5 μg plasmid DNA. After 4 h, 12.5% FBS was added and cells were incubated in T25 flasks overnight. After 24 h, selection was initiated by addition of 2 mg/ml zeocin to generate a stable cell line. After 3–4 weeks of selection, cells were transferred to shaker flasks and production scaled up. Cells were harvested after 4 days of expression by centrifugation (1000 *g*, 15 °C, 15 min). ISG65-containing supernatant was subsequently cleared from debris by centrifugation (13,000*g* 15 °C, 10 min.) and filtration (0.22 μm). Using tangential flow filtration, the cleared supernatant was concentrated and exchanged to buffer E (20 mM Tris pH7.5, 100 mM NaCl). The protein concentrate was loaded onto a CaptureSelect C-tag affinity column (ThermoFischer Sci.) using an ÄKTA start. After washing with buffer E, C-tagged ISG65 was specifically eluted with buffer E supplemented with 2 M MgCl_2_.

### Circular dichroism spectroscopy

For ISG65, far-UV CD experiments were carried out on a Jasco J-1500 spectropolarimeter with a 0.2 mm path cell. Protein was measured at a concentration of 0.4 mg ml^−1^ in buffer F (20 mM HEPES pH 7.5, 150 mM NaF). Spectra were recorded between 195 and 260 nm wavelength at an acquisition speed of 10 nm min^−1^ and corrected for buffer absorption. For calculation of melting curves at 222 nm, spectra were recorded every 5° between 5 ° and 80C with a slope of 0.16 °C min^−1^. During measurements the temperature was kept constant.

In both cases, the raw CD data (ellipticity θ in mdeg) were normalized for the protein concentration and the number of residues according to the equation below, yielding the mean residue ellipticity ([θ] in deg cm^2^ mol^−1^), where *MM*, *n*, *C*, and *l* denote the molecular mass (Da), the number of amino acids, the concentration (mg mL^−1^), and the cuvette path length (cm), respectively.

$$\left[ {\uptheta } \right] = \frac{{{\uptheta } \cdot MM}}{n \cdot C \cdot l}$$

Secondary structure content was calculated using the DichroWeb server^27^.

### Hydrogen–deuterium exchange mass spectrometry

#### Peptide mapping

300 pmol of ISG65 were mixed in 1:1 (v/v) ratio with 1 M glycine, 400 mM Tris(2-carboxyethyl)phosphine (TCEP) at pH 2.3 and injected onto a Nepenthesin-2 column. Generated peptides were trapped and desalted using a micro-trap column (Luna Omega 5 μm Polar C18 100 Å Micro Trap 20 × 0.3 mm) for 3 min at a flow rate 400µL min^-1^ using an isocratic pump delivering 0.4% formic acid in water. Both protease column and trap column were placed in an icebox. After 3 min, peptides were separated on a C18 reversed-phase column (Luna Omega 1.6 μm Polar C18 100 Å, 100 × 1.0 mm) with a linear gradient of 5–35% B over 26 min, where solvent A was 2% acetonitrile/0.4% formic acid in water and solvent B 95% acetonitrile/5% water/0.4% formic acid. The analytical column was placed in an icebox. A 15 T solariX XR FT-ICR mass spectrometer (Bruker Daltonics) operating in positive MS/MS mode was used for detection of peptides. Data was processed by DataAnalysis 4.2 software (Bruker Daltonics). MASCOT search engine was used for identification of peptides using a sequence of ISG65.

#### HDX

Hydrogen deuterium exchange was initiated by ten-fold dilution of ISG65 into a deuterated buffer. 50 µl aliquots (100 pmol) were taken after 20 s, 120 s, 1200 s and 7200 s of incubation in deuterated buffer, the reaction was quenched by addition of 50 µl of 1 M glycine, 400 mM TCEP at pH 2.3 and immediate freezing in liquid nitrogen. Aliquots were quickly thawed and analysed using the same system as described above. Peptides were separated over a linear gradient of 10–30% B over 18 min. The mass spectrometer was operated in positive MS mode. Spectra of partially deuterated peptides were processed via Data Analysis 4.2 (Bruker Daltonics, Billerica, MA) and the in-house program DeutEx (unpublished).

### Model prediction

Structure prediction was performed using the I-TASSER (Iterative Threading ASSEmbly Refinement) online server with default settings^23^.

## Supplementary Information

Supplementary Information.

## Data Availability

The datasets generated during and/or analysed during the current study are available from the corresponding author on reasonable request.
