# Supplementary material for: A multifaceted strategy to improve recombinant expression and structural characterisation of a Trypanosoma invariant surface protein
Source: Sci Rep. 2022 Jul 26;12:12706. doi: 10.1038/s41598-022-16958-x (PMC9325691; doi:10.1038/s41598-022-16958-x)
Supplement: Supplementary file 1 — Supplementary Information. [file 41598_2022_16958_MOESM1_ESM.pdf]

Supplementary information

A multifaceted strategy to improve recombinant expression and structural characterisation of a *Trypanosoma* invariant surface protein

Hagen Sülzen[1,2], Jitka Votrubova[1], Arun Dhillon[1] and Sebastian Zoll[1*]

[1]Institute of Organic Chemistry and Biochemistry, Academy of Sciences of the Czech Republic, Flemingovo namesti 2, 16610 Prague 6, Czech Republic

[2]Faculty of Science, Charles University, Albertov 6, 12800 Prague 2, Czech Republic

[*]Corresponding author: SZ, +420 220 183 464, sebastian.zoll@uochb.cas.cz

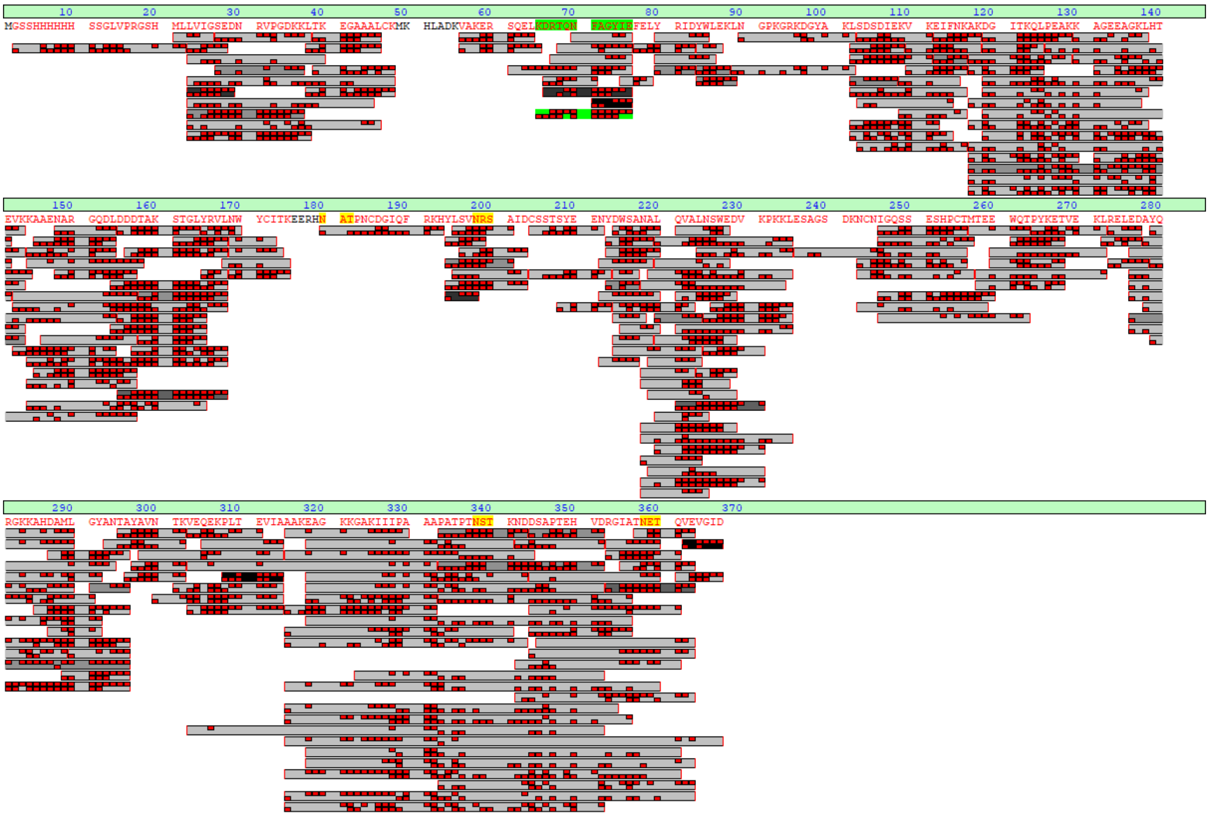

Supplementary Figure 1. ISG65 peptide mapping. In total 239 ISG65-derived peptides were identified. This corresponds to a sequence coverage of 97%. The covered sequence is depicted in red.

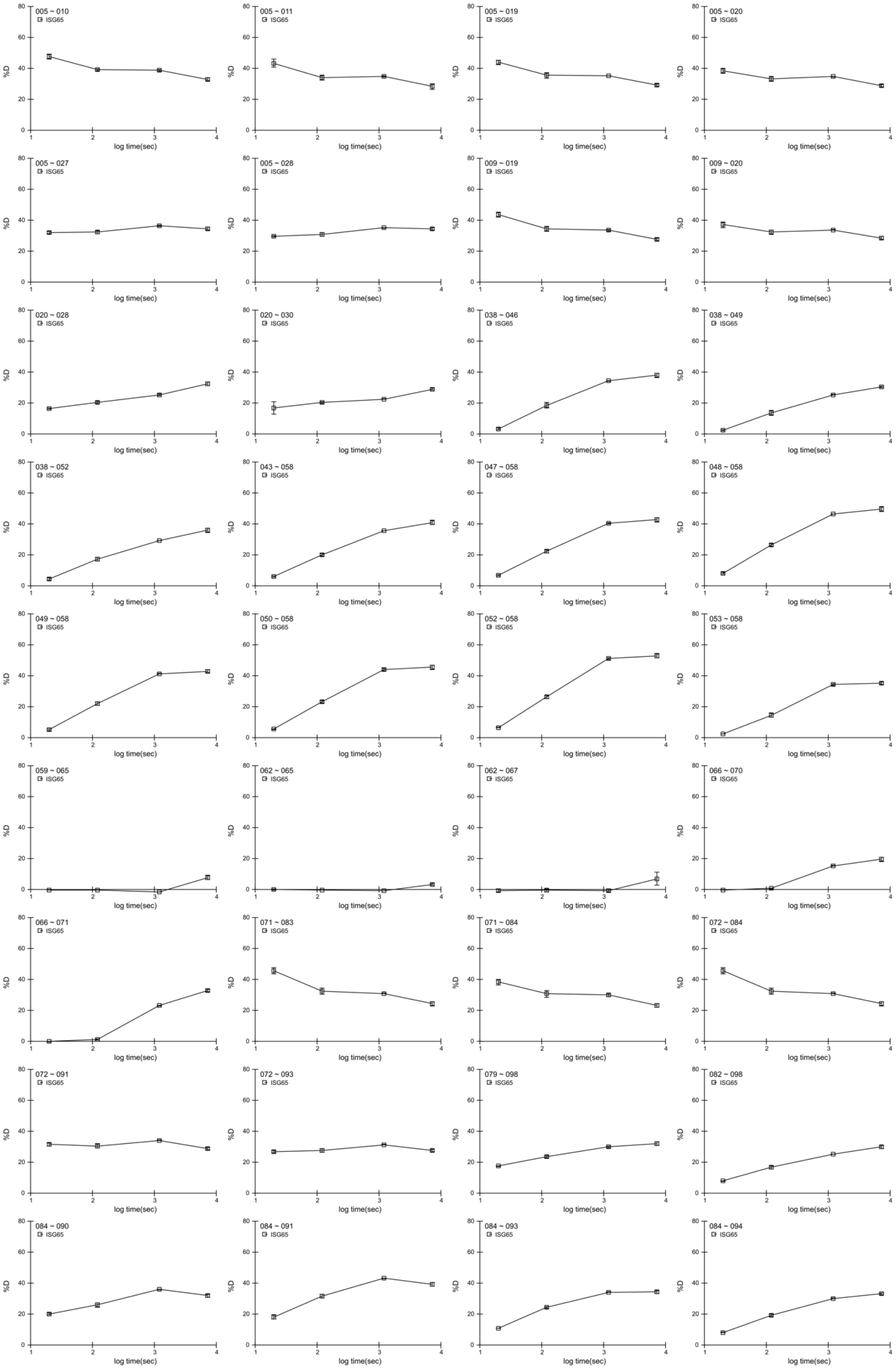
005 ~ 010
005 ~ 011
005 ~ 019
005 ~ 020
005 ~ 027
005 ~ 028
009 ~ 019
009 ~ 020
020 ~ 028
020 ~ 030
038 ~ 046
038 ~ 049
038 ~ 052
043 ~ 058
047 ~ 058
048 ~ 058
049 ~ 058
050 ~ 058
052 ~ 058
053 ~ 058
059 ~ 065
062 ~ 065
062 ~ 067
066 ~ 070
066 ~ 071
071 ~ 083
071 ~ 084
072 ~ 084
072 ~ 091
072 ~ 093
079 ~ 098
082 ~ 098
084 ~ 090
084 ~ 091
084 ~ 093
084 ~ 094
ISG65
%D
log time(sec)

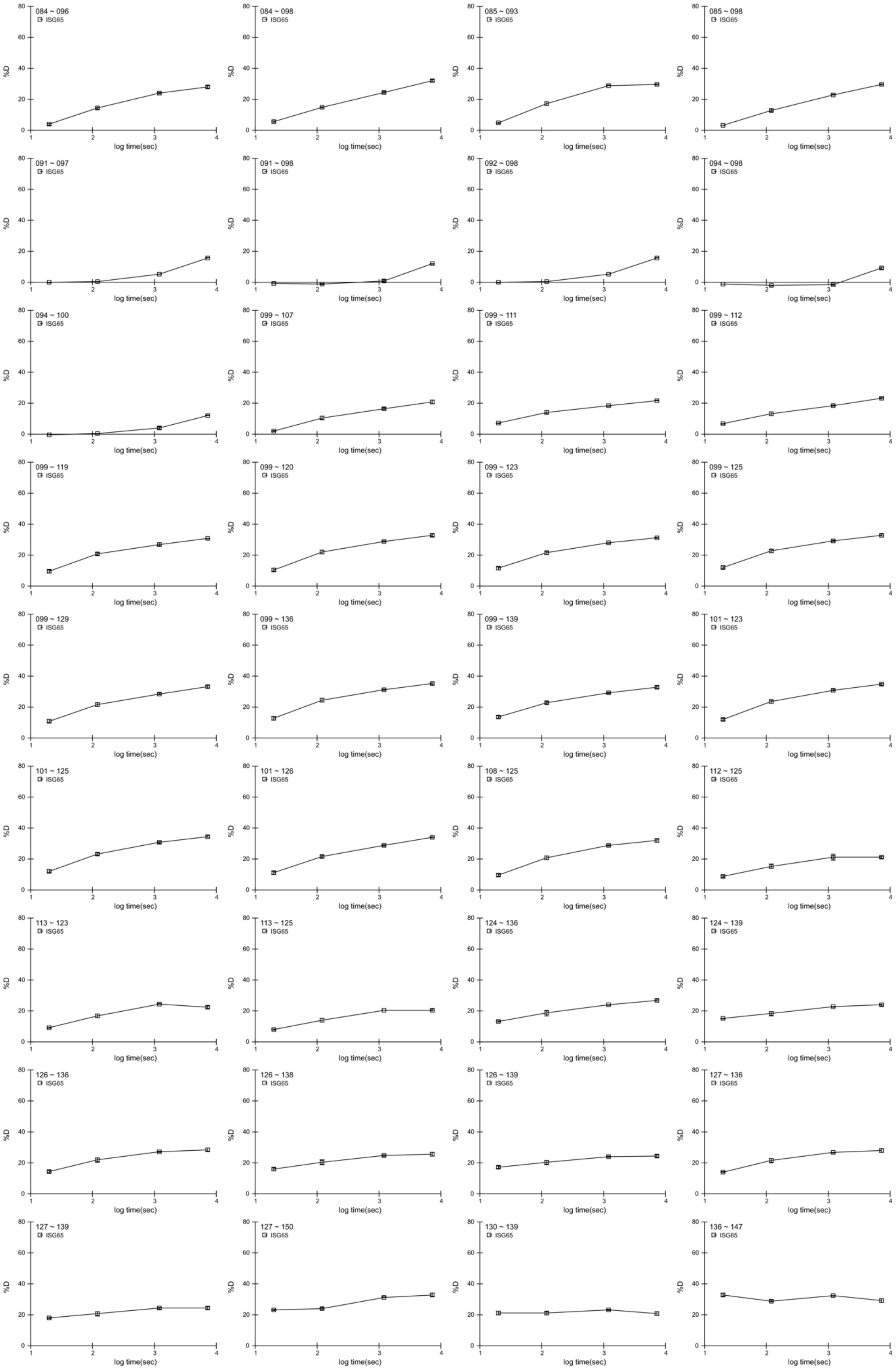
084 ~ 096
084 ~ 098
085 ~ 093
085 ~ 098
091 ~ 097
091 ~ 098
092 ~ 098
094 ~ 098
094 ~ 100
099 ~ 107
099 ~ 111
099 ~ 112
099 ~ 119
099 ~ 120
099 ~ 123
099 ~ 125
099 ~ 129
099 ~ 136
099 ~ 139
101 ~ 123
101 ~ 125
101 ~ 126
108 ~ 125
112 ~ 125
113 ~ 123
113 ~ 125
124 ~ 136
124 ~ 139
126 ~ 136
126 ~ 138
126 ~ 139
127 ~ 136
127 ~ 139
127 ~ 150
130 ~ 139
136 ~ 147
ISG65
%D
log time(sec)

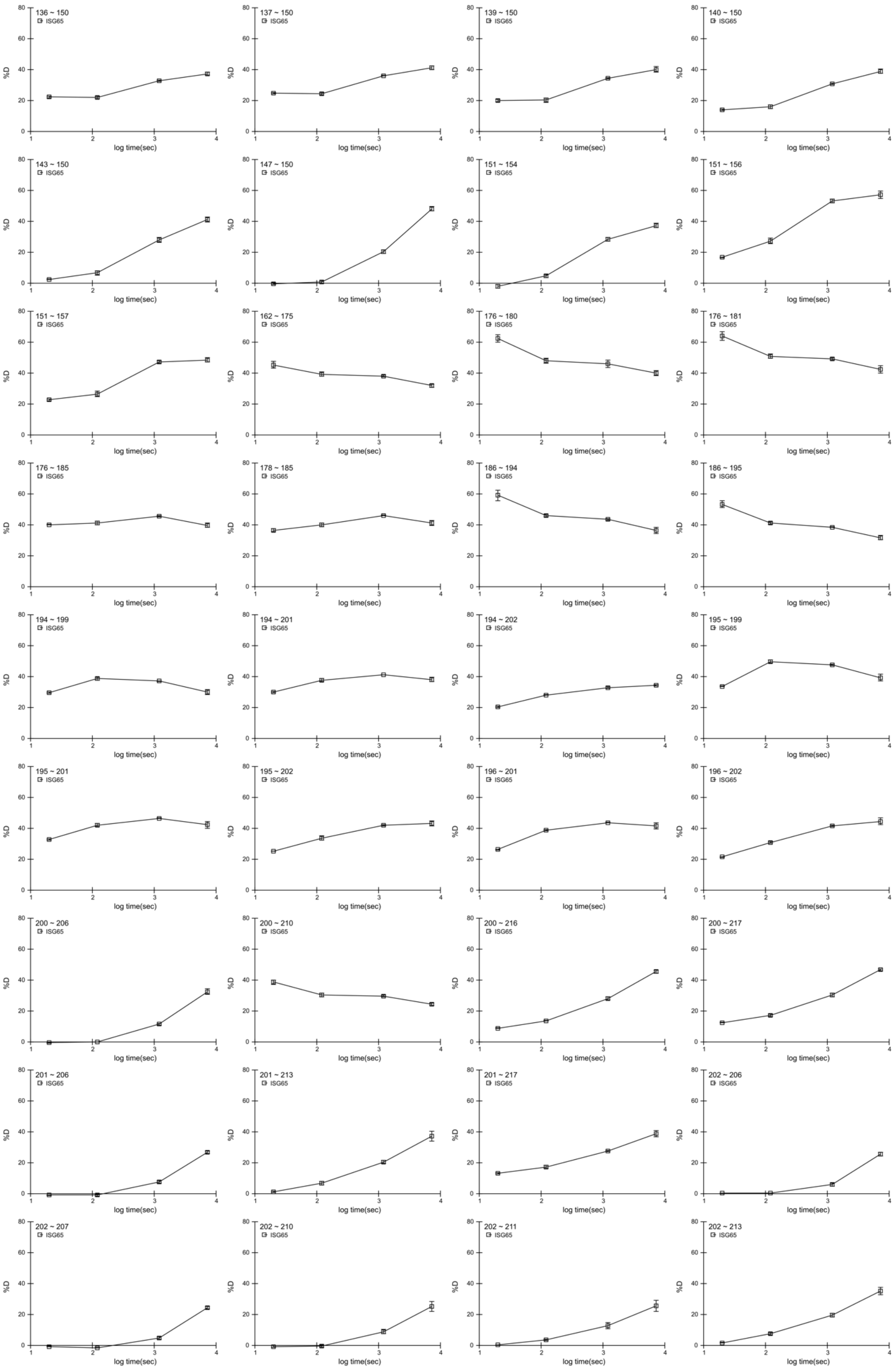
136 ~ 150
137 ~ 150
139 ~ 150
140 ~ 150
143 ~ 150
147 ~ 150
151 ~ 154
151 ~ 156
151 ~ 157
162 ~ 175
176 ~ 180
176 ~ 181
176 ~ 185
178 ~ 185
186 ~ 194
186 ~ 195
194 ~ 199
194 ~ 201
194 ~ 202
195 ~ 199
195 ~ 201
195 ~ 202
196 ~ 201
196 ~ 202
200 ~ 206
200 ~ 210
200 ~ 216
200 ~ 217
201 ~ 206
201 ~ 213
201 ~ 217
202 ~ 206
202 ~ 207
202 ~ 210
202 ~ 211
202 ~ 213
ISG65
%D
log time(sec)

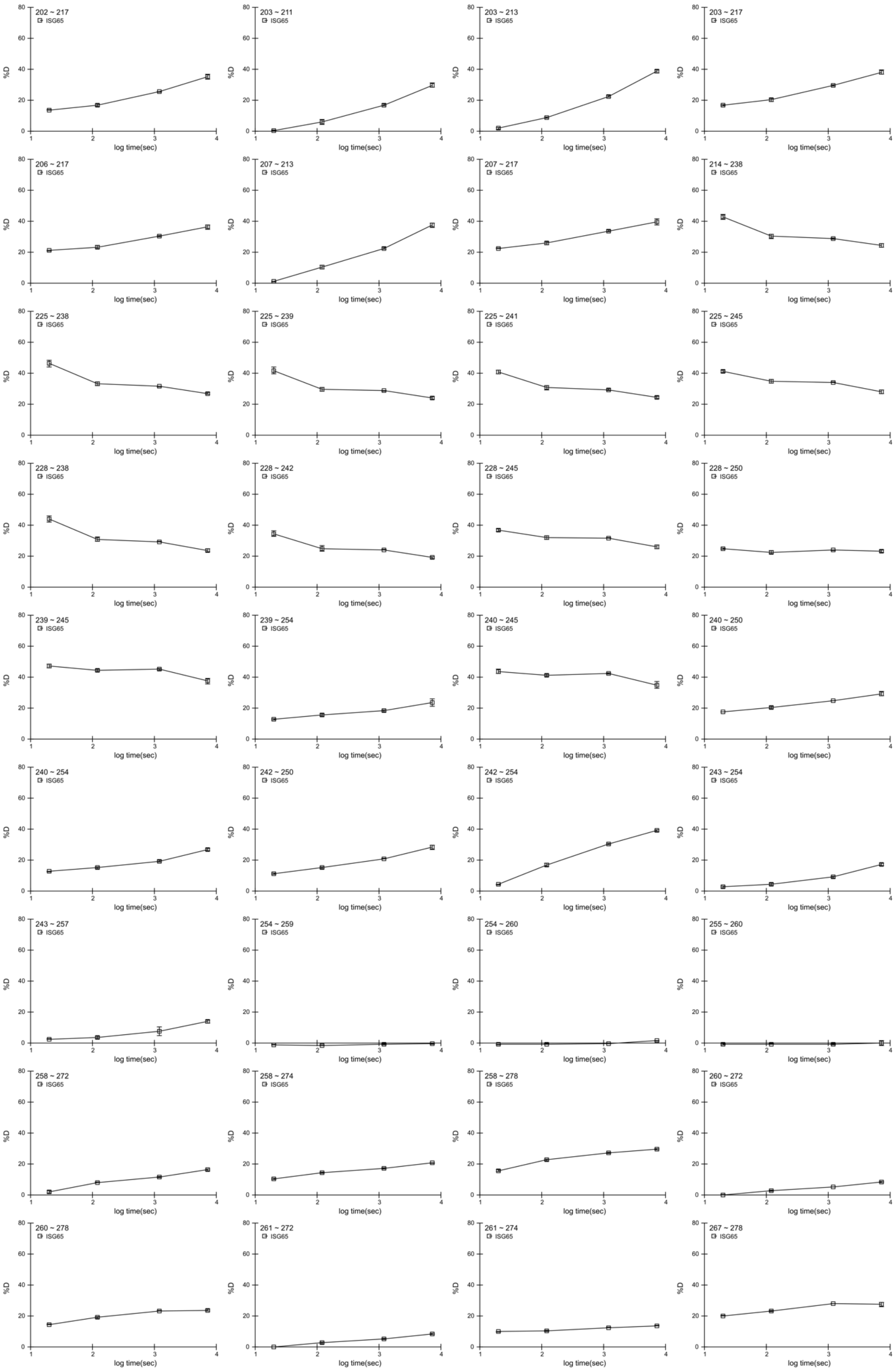
202 ~ 217
203 ~ 211
203 ~ 213
203 ~ 217
206 ~ 217
207 ~ 213
207 ~ 217
214 ~ 238
225 ~ 238
225 ~ 239
225 ~ 241
225 ~ 245
228 ~ 238
228 ~ 242
228 ~ 245
228 ~ 250
239 ~ 245
239 ~ 254
240 ~ 245
240 ~ 250
240 ~ 254
242 ~ 250
242 ~ 254
243 ~ 254
243 ~ 257
254 ~ 259
254 ~ 260
255 ~ 260
258 ~ 272
258 ~ 274
258 ~ 278
260 ~ 272
260 ~ 278
261 ~ 272
261 ~ 274
267 ~ 278
ISG65
%D
log time(sec)

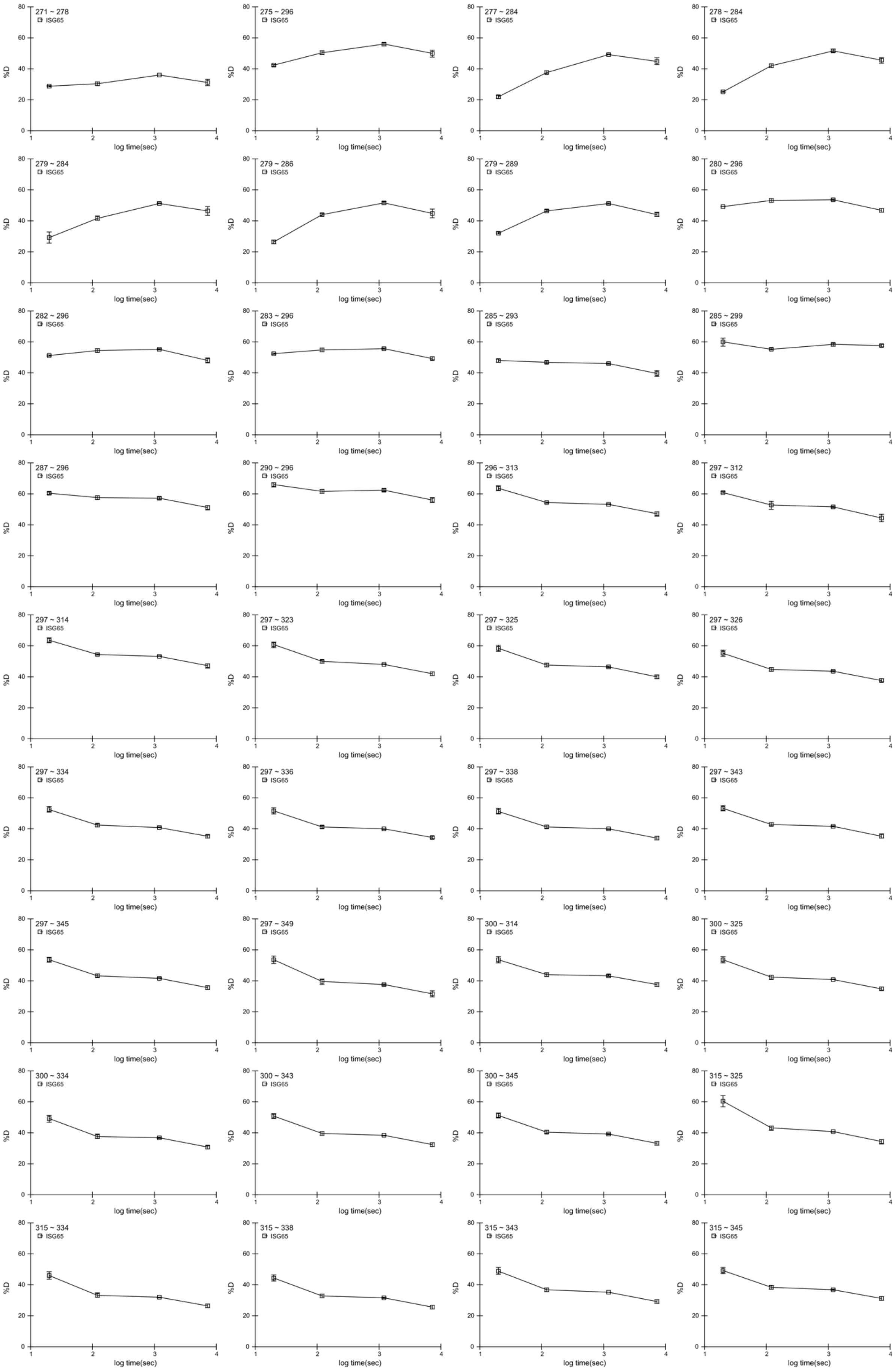
271 ~ 278
275 ~ 296
277 ~ 284
278 ~ 284
279 ~ 284
279 ~ 286
279 ~ 289
280 ~ 296
282 ~ 296
283 ~ 296
285 ~ 293
285 ~ 299
287 ~ 296
290 ~ 296
296 ~ 313
297 ~ 312
297 ~ 314
297 ~ 323
297 ~ 325
297 ~ 326
297 ~ 334
297 ~ 336
297 ~ 338
297 ~ 343
297 ~ 345
297 ~ 349
300 ~ 314
300 ~ 325
300 ~ 334
300 ~ 343
300 ~ 345
315 ~ 325
315 ~ 334
315 ~ 338
315 ~ 343
315 ~ 345
ISG65
%D
log time(sec)

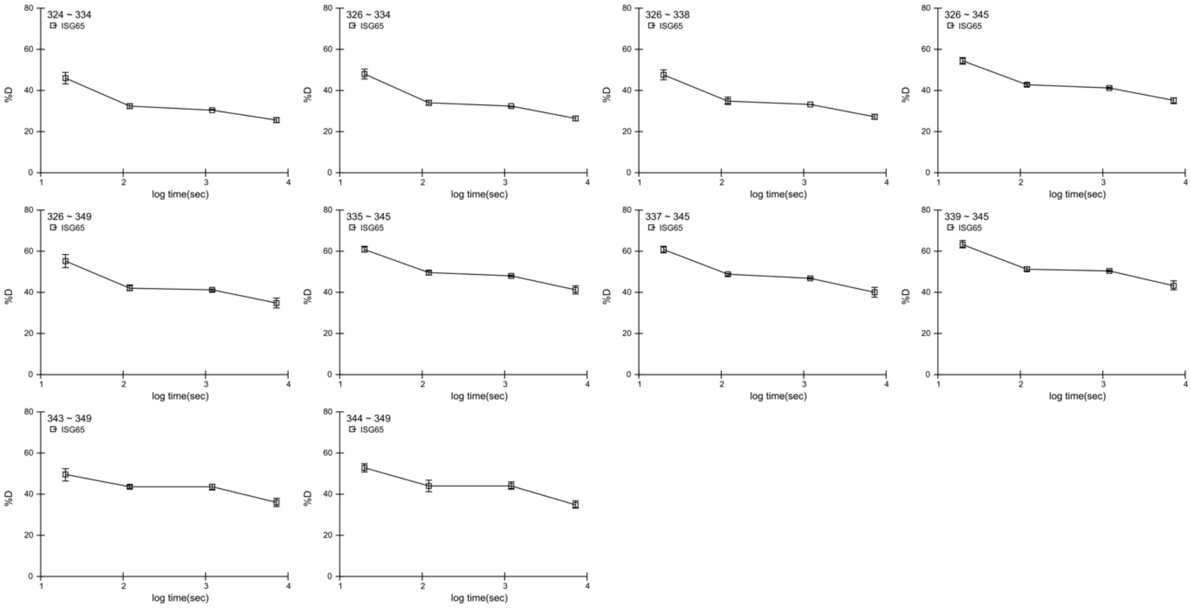

Supplementary Figure 2. Rates of deuterium uptake for ISG65-derived peptides. Peptide masses were determined after 20s, 120s and 1200s and 7200s and the percentage of deuterium uptake calculated. The position of each peptide in the sequence of ISG65 is indicated. The HDX-MS experiments were performed in triplicates.

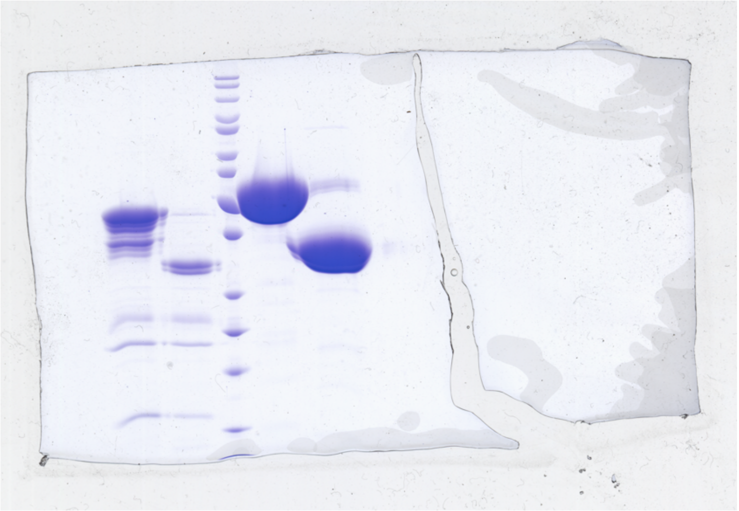

Supplementary Figure 2. Complete SDS separating gel as shown digitally cropped in Figure 3A.

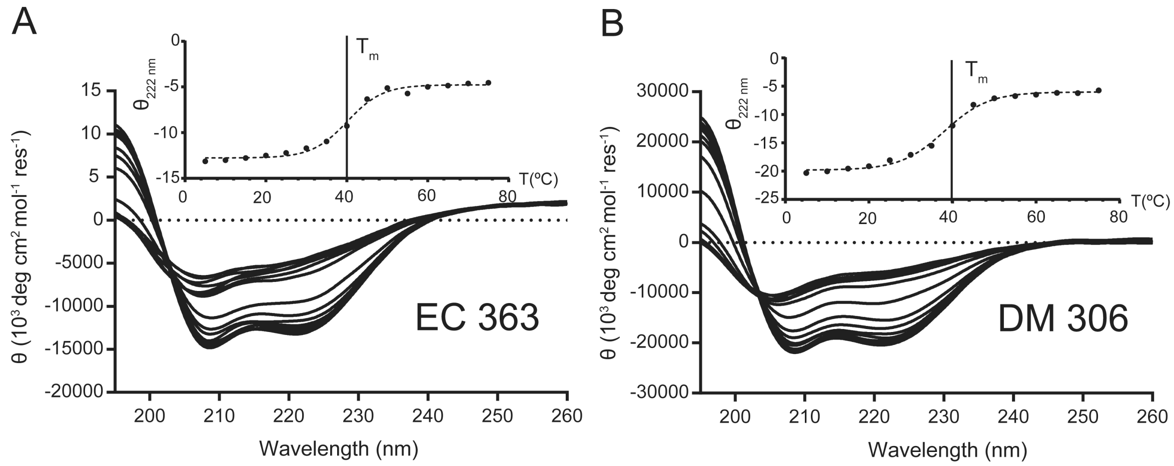

Supplementary Figure 4. Longest and shortest ISG65 construct have different α-helical content but similar melting temperatures. (A) CD spectra collected between 5°C and 80°C and melting curve of construct $ISG65_{18\text{-}363}$, expressed in *E. coli* (EC). **(B)** CD spectra collected between 5°C and 80°C and melting curve of construct $ISG65_{32\text{-}306}$, expressed in *Drosophila melanogaster* (DM) S2 cells. Melting temperatures (T_m) were calculated from mean residue ellipticity (θ) measured at 222nm. Filled circles represent the experimental data points and dashed lines the fit.

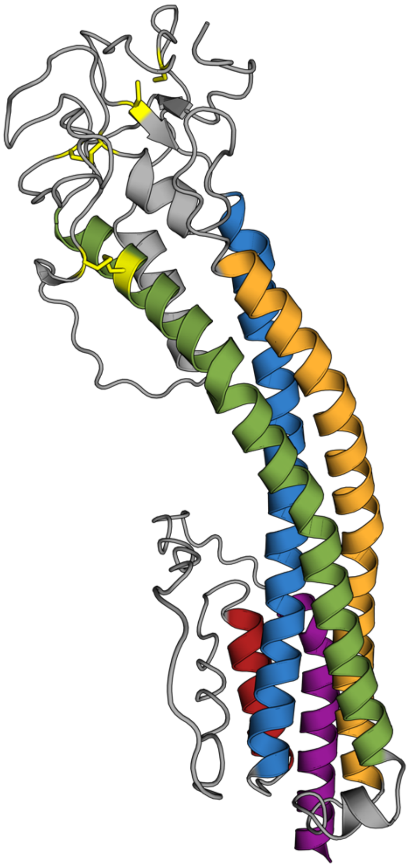

Supplementary Figure 5. Model of ISG65, predicted by AlphaFold2 for Trypanosoma and Leishmania proteins. Structure prediction of $ISG65_{18\text{-}387}$ by AlphaFold2 (adapted for *Trypanosoma* and *Leishmania)* [1]. Helices forming the three-helix bundle canonical for structurally characterised, GPI-anchored trypanosoma surface proteins are highlighted in orange, blue and green (ref. Fig 4). Two predicted C-terminal helices, connected by a long, disordered region are highlighted in red and purple, respectively. Cysteines are highlighted in yellow.

Supplementary References

1. Wheeler, R.J., *A resource for improved predictions of Trypanosoma and Leishmania protein three-dimensional structure.* PLOS ONE, 2021. **16**(11).
